# Unnecessary Noise

**Published:** 1920-11-13

**Authors:** 


### UNNECESSARY NOISE.

We have heard a good deal recently of the various factors which daily tend to disturb the mental and physical equilibrium of the modern public, but leaving aside the host of social problems which immediately come to mind, it seems to the casual medical observer that one of the most insidious, particularly in the large cities of to-day, is the wholesale production of unnecessary noise.

It is true that sundry irritating varieties have at various times been officially stamped out. But they are as nothing to the gradually but greatly increased volume of sound accompanying most of our methods and means of transport. Thus have been produced many kinds and variations of auditory stimuli. It may be impossible especially to condemn any one of them for direct harm that it causes, for it is an accepted fact that noise which is aggravating to one individual may be almost pleasurable to another. But it is nevertheless certain that the combined effect of the din which commonly surrounds the town dweller during his waking, and often his sleeping, hours must have injurious effects that are, generally speaking, as unnecessary as they are incalculable.

From the purely medical standpoint we may well compare the effect of noise upon the susceptible individual with that of the chronic and cumulative effect of blows delivered upon a weakened or diseased part of the human body. In the great average, this weakness may be but the mildest form of nervous disorder. But the result may be ultimately out of all proportion to the initial cause.

It is an axiom in hospital practice that noise is a direct danger to the sick, and particularly to the disordered nervous mechanism. Not only does the notice near the approach to a hospital, or the occasional prevention of traffic uproar near the sick man's house, ensure that, in the obvious instance, the validity of our plea is publicly recognised, but also it must be equally clear that the relative success and comfort of quiet nursing homes, sanatoria, and the like have borne eloquent testimony to the beneficial results to be secured and the vital importance of moderating as much as possible the myriad noises which attend our daily life.

To us it seems that elimination of unnecessary noise is of such pressing concern to the public in general that there is an urgent call for action by the civil authorities. If they require evidence of this need we are convinced that the highest medical opinion will readily provide it. But at the same time we must admit that no single set of immediate measures can be expected to deal with a progressive evil which has already advanced so far. There is no reason, however, why a determined campaign of noise reduction should not be commenced. Several controlling concerns in heavy motor road transport are, we are glad to note, taking no little pains to reduce the uproar coming from this particular source. But there are thousands of utterly neglected instances. The effect of a successful anti-noise campaign might not prolong the life of material structures, as did the attack on the smoke nuisance, but it would contribute in no small degree to the health and welfare of the urban public.

